# Regulation of Reentrainment Function Is Dependent on a Certain Minimal Number of Intact Functional ipRGCs in rd Mice

**DOI:** 10.1155/2017/6804853

**Published:** 2017-11-22

**Authors:** Jingxue Zhang, Huaizhou Wang, Shen Wu, Qian Liu, Ningli Wang

**Affiliations:** ^1^Beijing Institute of Ophthalmology, Beijing Tongren Eye Center, Beijing Ophthalmology & Visual Sciences Key Laboratory, Beijing Tongren Hospital, Capital Medical University, Beijing, China; ^2^Beijing Tongren Eye Center, Beijing Ophthalmology & Visual Sciences Key Laboratory, Beijing Tongren Hospital, Capital Medical University, Beijing, China

## Abstract

**Purpose:**

To investigate the effect of partial ablation of melanopsin-containing retinal ganglion cells (mcRGCs) on nonimage-forming (NIF) visual functions in rd mice lacking rods.

**Methods:**

The rd mice were intravitreally injected with different doses (100 ng/*μ*l, 200 ng/*μ*l, and 400 ng/*μ*l) of immunotoxin melanopsin-SAP. And then, the density of ipRGCs was examined. After establishing the animal models with different degrees of ipRGC damage, a wheel-running system was used to evaluate their reentrainment response.

**Results:**

Intravitreal injection of melanopsin-SAP led to partial ablation of ipRGCs in a dose-dependent manner. The survival rates of ipRGCs in the 100 ng/*μ*l, 200 ng/*μ*l, and 400 ng/*μ*l groups were 74.14% ± 4.15%, 39.25% ± 2.29%, and 38.38% ± 3.74%, respectively. The wheel-running experiments showed that more severe ipRGC loss was associated with a longer time needed for reentrainment. When the light/dark cycle was delayed by 8 h, the rd mice in the PBS control group took 4.67 ± 0.79 days to complete the synchronization with the shifted cycle, while those in the 100 ng/*μ*l and 200 ng/*μ*l groups required 7.90 ± 0.55 days and 11.00 ± 0.79 days to complete the synchronization with the new light/dark cycle, respectively.

**Conclusion:**

Our study indicates that the regulation of some NIF visual functions is dependent on a certain minimal number of intact functional ipRGCs.

## 1. Introduction

Mammalian eyes mediate both image-forming (IF) and nonimage-forming (NIF) visual functions. NIF vision provides a measure of the ambient light for the purposes of synchronization of circadian clocks to light/dark cycles and regulation of pupil size, pineal melatonin production, and other functions. People traditionally believe that the classical photoreceptors (rods and cones) regulate both IF and NIF visual pathways. But recently, a small subset of retinal ganglion cells (RGCs) called intrinsically photosensitive RGCs (ipRGCs) or melanopsin-containing RGCs (mcRGCs), projecting to the suprachiasmatic nucleus and other pretectal areas, has been identified to be a third type of mammalian photoreceptors and determined to be photosensitive [1–3]. The discovery of ipRGCs has allowed for rapid progress in the past decade toward understanding the NIF visual system, especially the three types of photoreceptors [4–13].

Previous studies have attempted to elucidate the respective roles of these photoreceptor types in NIF visual functions, most of which showed that NIF vision was almost not affected after the degeneration of rods and cones [14–17]. Melanopsin-knockout mice (opn4^−/−^) retained generally normal NIF functions expect for an attenuated phase-shifting response to light and a diminished pupillary constriction at high irradiance levels. In addition, the effect of light on circadian rhythm disappeared after the loss of both melanopsin and rods and cones [18–21]. It therefore seems that NIF visual functions can be supported by photoreception in either rods and cones or ipRGCs.

It should be noted that most of the available data comes from studies using animals carrying genetic lesions. These studies only abolished the genetic expression of melanopsin, while the functions of ipRGCs as ganglion cells remained unchanged, especially the projection to brain areas and the transduction of photic information. In order to fully clarify the roles of ipRGCs, it is imperative to ablate the whole cell rather than the expression of melanopsin alone. Recent studies reported that after targeted destruction of ipRGCs, all of the light-regulated NIF functions were dramatically impaired or even disappeared [22–24]. This indicates that in addition to their intrinsic photosensitivity, ipRGCs also function as a conduit through which rods and cones can access brain circuits mediating NIF functions. Thus, ipRGCs seem to play a unique role in NIF visual responses.

In the above-mentioned studies, however, as the ablation of ipRGCs occurred in the presence of rods and cones, there was a possibility that the signals generated by the rods and cones might be carried to the brain to mediate NIF functions through other pathways. So the respective roles of the three types of photoreceptors, that is, ipRGCs, rods, and cones, in multiple NIF functions cannot be clearly established.

Moreover, in prior studies, only partial ablation of ipRGCs already led to impairment of NIF functions, suggesting that a minimal number of ipRGCs may be required for NIF visual responses. Thus, our study aimed to clarify and quantify the specific contributions of ipRGCs to NIF functions. In order to exclude the possible auxiliary functions of rods and cones, we selected rd mice (C3H/Hej) lacking rods and cones. Animal models with different degrees of ipRGC damage were established via intravitreal injection of different doses of immunotoxin melanopsin-SAP. And then, a wheel-running system was used to evaluate the reentrainment function (one of the most important NIF functions) of these animals. Based on the changes in the reentrainment response, we attempted to elucidate the roles of ipRGCs in NIF functions and determine whether the regulation of such functions is dependent on a certain number of ipRGCs.

## 2. Materials and Methods

### 2.1. Animals

C3H/HeJ male mice (Jackson Laboratories, Bar Harbor, ME, USA) were used in this study. These mice were homozygous for the retinal degeneration 1 mutation (Pde6b rd1), which caused blindness by weaning age. All experimental and animal care procedures were strictly in accordance with the institutional guidelines and the ARVO Statement for the Use of Animals in Ophthalmic and Vision Research. The study protocol was approved by the Animal Care and Use Committee at the Capital Medical University (permit number 2010-X-30). All surgery was performed under sodium pentobarbital anesthesia, and all efforts were made to minimize suffering.

### 2.2. Comparison of the Number of ipRGCs in rd Mice at Different Ages

In order to determine whether all damages to ipRGCs were caused by the injection of immunotoxin, or whether theses damages were also potentially related with age, we immunohistochemically labeled ipRGCs using antibodies against melanopsin and compared the number of ipRGCs on flat-mounted retina among 1-, 3-, and 6-month-old rd mice (*n* = 6 in each age group) (the detailed methodology is described below).

### 2.3. Eye Injections

Three-month-old C3H/HeJ mice were divided into 3 dose groups (*n* = 6 in each group): 100 ng/*μ*l, 200 ng/*μ*l, and 400 ng/*μ*l. All animals were anesthetized with 5% chloraldurat (8 *μ*l/g), and the eyes were topically anesthetized with one drop of 0.5% proparacaine (Alcon Laboratories Inc., Fort Worth, TX, USA). The left eye of each animal was intravitreally injected with different doses of immunotoxin melanopsin-SAP (2 *μ*l/eye) (Advanced Targeting Systems, San Diego, CA, USA), consisting of saporin conjugated to a melanopsin polyclonal antibody. A Hamilton syringe with a 30 gauge needle (BD Medical Systems, Franklin Lakes, NJ, USA) was used for the intravitreal injections, and the needle was left in place for about 3 minutes after the injections. The right eye of each animal was injected with 2 *μ*l phosphate-buffered saline (PBS) vehicle as control. All animals were sacrificed by CO_2_ asphyxiation 4 weeks after injection. The sampling time of each mouse was fixed at 14:00. The density of ipRGCs was examined on flat-mounted retina.

### 2.4. Retinal Processing and Staining

The procedures of retinal processing and staining were performed as described in our previous study [25]. Briefly, for flat mount, a slit was cut in the sclera close to the cornea. The eyes were then submerged in 0.01 mol/l PBS. The front part (cornea, lens, and vitreous) of the eye was cut away, and the retina was carefully isolated from the pigment epithelium. The retinas were fixed in fresh 4% paraformaldehyde in PBS for 30 minutes and then washed three times in PBS for 5 minutes each. The free-floating retinas were incubated in a blocking solution (0.3% Triton X-100 and 5% bovine serum albumin in PBS) for 1 hour at room temperature and were then incubated with a primary melanopsin antibody (polyclonal rabbit anti-melanopsin; Affinity BioReagents, Golden, CO, USA) at 1:500 dilution in PBS/0.3% Triton X-100/5% bovine serum albumin for 72 hours at 4°C. After three washes in PBS of 15 minutes each, the fluorescence-conjugated secondary antibody (Alexa Fluor 488 goat antibody to rabbit immunoglobulin G; Molecular Probes, Eugene, OR, USA) was applied to the sample as previously described, except that incubation was for 2 hours at room temperature. The free-floating retinas were washed again as described above and flat mounted onto glass slides, and coverslips were applied using Vect Mount Permanent Mounting Medium (Vector Laboratories, Burlingame, CA, USA).

For Hematoxylin and Eosin (H&E) staining, the eyes were removed as above and the eyecups were fixed in 4% paraformaldehyde for 2 hours, washed three times with PBS, and embedded in paraffin. Eight-micron-thick paraffin sections were used for H&E staining. Stained slides were visualized under a Leica microscope. The thickness of the inner nuclear layer (INL) was measured using image pro plus (IPP) software.

### 2.5. Counting of ipRGCs

When counting cell number, we chose 8 visual fields for each flat-mounted retina as described in our previous study [25]. The melanopsin-positive cells (ipRGCs) were counted at 200x magnification using a confocal microscope (Leica TCS SP2, Leica Microsystems, Heidelberg, Germany). Each retina was counted in a double-blind manner. The ipRGC survival rate was defined as (ipRGC number of experimental eye)/(ipRGC number of control eye) × 100%.

### 2.6. Wheel-Running Experiments

A wheel-running system (Mini Mitter Co. Inc., Sunriver, OR, USA) was used to evaluate the NIF visual function of the rd mice in the PBS control group (*n* = 3), 100 ng/*μ*l group (*n* = 5), and 200 ng/*μ*l group (*n* = 5), respectively. C57 mice were involved as wild-type control (*n* = 3). Since the survival rate of ipRGCs in the 400 ng/*μ*l group was similar to that in the 200 ng/*μ*l group, the rd mice in the 400 ng/*μ*l group were not included in the wheel-running experiments.

When rhythms were stable, each mouse was deeply anesthetized and unilaterally enucleated, and then the remaining eye was injected intravitreally with different doses of immonotoxin as mentioned above. After the injections, animals were allowed to recover in 12 h light: 12 h dark conditions.

Each mouse was placed in a cage with a running wheel inside an enclosure in which white LED lighting was computer controlled and the irradiance was about 100 lux. Each enclosure contained 8 mouse cages. Wheel revolutions were monitored by computer, stored as revolutions/min, then summed across each 5 min interval, and plotted in raster format using ActiView (Mini Mitter Co. Inc., Sunriver, OR, USA) software.

When entrainment stabilized, in order to assess the adjustment function of the circadian clock to light conditions (reentrainment), an important NIF charged by ipRGCs, mice were subjected to a jet lag test with 8 h delay in the time of lights on and lights off to evaluate whether they could resynchronize with the shifted cycle. The light condition was still 12 h light: 12 h dark. After the 8 h delay of the LD cycle, the number of days to reentrain was defined for each animal as the number of days required to shift activity midpoint by 8 ± 0.25 h. The reentrainment was analyzed using the ActiView software.

### 2.7. Statistical Analysis

All data were expressed as mean ± SD (standard deviation). An independent sample *t*-test was used to compare the differences in the mean INL thickness. One-way ANOVA with Tukey's multiple comparison test was used to compare the differences in the mean number of ipRGCs and the survival rate of ipRGCs among different groups. For behavioral analysis, we used two-way ANOVA, followed by Fisher's LSD post hoc tests. *P* < 0.05 was considered statistically significant.

## 3. Results

### 3.1. Number of ipRGCs in rd Mice at Different Ages

**Figure 1:**
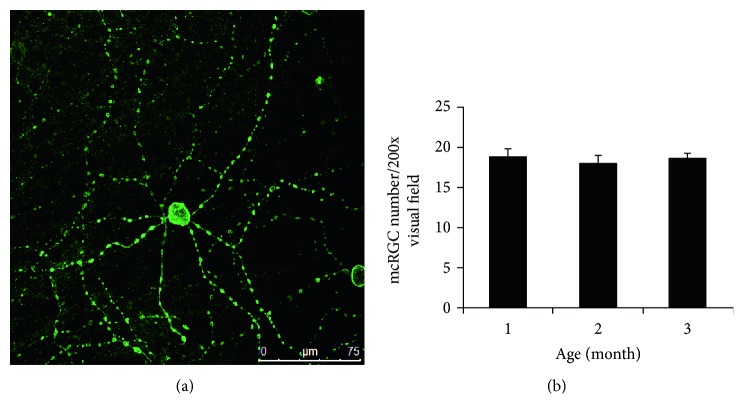
The ipRGCs could be immunohistochemically labeled using antibodies against melanopsin. (a) The laser scanning confocal view of ipRGCs in flat-mounted retina, showing melanopsin immunoreactivity and cell morphology. Bar = 75 *μ*m. (b) The bar graph comparing the numbers of ipRGCs per visual field (200x magnification) among the rd mice at different ages. The differences in the number of ipRGCs were not statistically significant (one-way ANOVA with Tukey's multiple comparison test, *P* > 0.05).

The ipRGCs were immunohistochemically labeled using antibodies against melanopsin (Figure 1(a)). It was observed that the ipRGCs, having a diameter of about 20 *μ*m, were diffusely distributed throughout the retina, with 3–5 dendritic branches per cell, a dendritic field diameter of about 300 *μ*m, manifesting a bead-like structure. No significant differences were noted in the distribution and morphology of ipRGCs among the rd mice at different ages. The numbers of ipRGCs per visual field at 200x magnification were counted to be 18.66 ± 1.10 in 1-month-old rd mice, 17.92 ± 1.00 in 3-month-old rd mice, and 18.51 ± 0.78 in 6-month-old rd mice. The differences in the number of ipRGCs were not statistically significant among the rd mice at different ages (all *P* > 0.05) (Figure 1(b)).

### 3.2. Survival Rate of ipRGCs after Immunotoxin Injection

**Figure 2:**
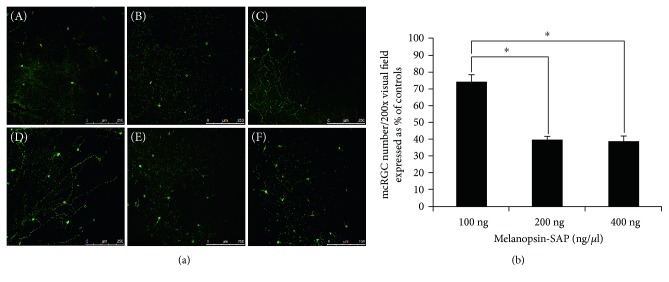
Intravitreal injection of melanopsin-SAP led to partial ablation of ipRGCs in a dose-dependent manner. (a) Immunohistochemically labeled ipRGCs using antibodies against melanopsin and comparison of the number of ipRGCs on flat-mounted retina following intravitreal injection of immunotoxin melanopsin-SAP in rd mice. (A–C) The number of ipRGCs per visual field (200x magnification) on flat-mounted retinas in different dose groups; (D–F) the results of the control eyes (PBS injection group). As the dose of melanopsin-SAP increased, the number of melanopsin-positive cells in the experimental eye decreased, while that in the control eye remained the same. Bar = 250 *μ*m. (b) Analysis of the survival rate of ipRGCs after immunotoxin injection. The ipRGC survival rate was defined as (ipRGC number of experimental eye)/(ipRGC number of control eye) × 100%. The survival rate of ipRGCs was significantly reduced in the 200 ng/*μ*l and 400 ng/*μ*l groups when compared with the 100 ng/*μ*l group (one-way ANOVA with Tukey's multiple comparison test, *P* < 0.01). But the difference between the 200 ng/*μ*l group and the 400 ng/*μ*l group was not statistically significant (one-way ANOVA with Tukey's multiple comparison test, *P* = 0.933). ^∗^*P* < 0.01.

When the flat-mounted retinas of different dose groups were compared, we observed a dose-dependent cell death of ipRGCs following intravitreal injection of immunotoxin melanopsin-SAP in rd mice. As the dose of melanopsin-SAP increased, the number of melanopsin-positive cells in the experimental eye decreased, while that in the control eye remained the same (Figure 2(a)). The survival rates of ipRGCs in the 100 ng/*μ*l, 200 ng/*μ*l, and 400 ng/*μ*l groups were 74.14% ± 4.15%, 39.25% ± 2.29%, and 38.38% ± 3.74%, respectively. The survival rate of ipRGCs was significantly reduced in the 200 ng/*μ*l and 400 ng/*μ*l groups when compared with the 100 ng/*μ*l group (both *P* < 0.01). But the difference between the 200 ng/*μ*l group and the 400 ng/*μ*l group was not statistically significant (*P* = 0.933) (Figure 2(b)).

### 3.3. INL Thickness

**Figure 3:**
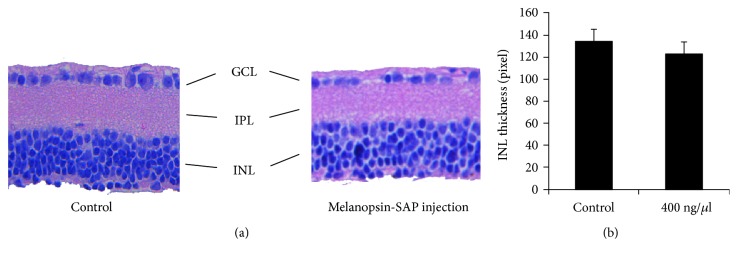
The morphological examination of retinal sections after injection of melanopsin-SAP immunotoxin in rd mice. (a) Photomicrographs of 8 *μ*m paraffin sections of retina were used for H&E staining. No significant morphological differences were observed between the highest dose group and the control group. Bar = 100 *μ*m. (IPL = inner plexiform layer; INL = inner nucleus layer; GCL = ganglion cell layer). (b) The thickness of the INL was measured using IPP software (pixel). The difference in the INL thickness between the highest dose group and the control group was not statistically significant (independent sample *t*-test, *P* > 0.05).

In order to determine whether the injection of immunotoxin melanopsin-SAP had any other effect on the retina, we compared the thickness of the inner nuclear layer (INL) of the retina between the highest dose (400 ng/*μ*l) group and the control group. Based on the H&E stained slides, the morphology of the retina was found to be similar between the two groups (Figure 3(a)); nearly all photoreceptors in the outer nuclear layer (ONL) disappeared, while the structure from the INL to the ganglion cell layer (GCL) remained intact. The results of the image pro plus (IPP) software analysis showed that the INL thickness of the control group was 133.40 ± 11.61 pixels and that of the highest dose group was 122.00 ± 9.70 pixels; the difference was not statistically significant (*P* > 0.05) (Figure 3(b)).

### 3.4. Wheel-Running Experiments

**Figure 4:**
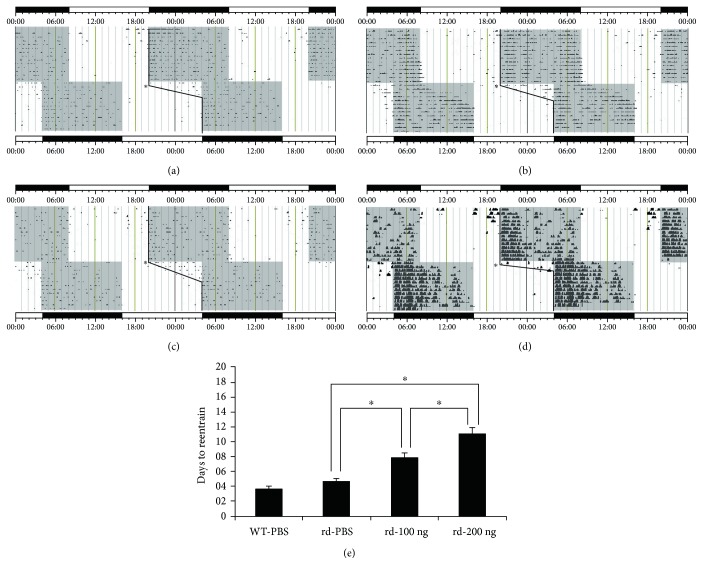
Entrainment and wheel-running periods of rd mice after injection of melanopsin-SAP immunotoxin. The bar below the actograms indicates the light (white) and dark (black) periods, and the light and dark periods are indicated by white and gray backgrounds, respectively. When the light/dark cycle was delayed by 8 h, (a) the rd mice in the PBS control group were capable of reentraining to the light/dark cycle, and they took 4.67 ± 0.79 days to complete the synchronization with the shifted cycle. (b) The rd mice in the 100 ng/*μ*l group and (c) the rd mice in the 200 ng/*μ*l group required 7.90 ± 0.55 days and 11.00 ± 0.79 days to complete the synchronization with the new light/dark cycle, respectively, indicating slower reentrainment. (d) The wild-type mice took 3.67 ± 0.29 days to complete the synchronization with the shifted cycle, and the locomotor activity of the wild-type mice was more robust than that of the rd mice. ^∗^ represents the day delay in the time of lights on and lights off. (e) The differences in the number of days needed for reentrainment were not statistically significant between the rd mice in the PBS control group and the wild-type group (two-way ANOVA, followed by Fisher's LSD post hoc test, *P* > 0.05). The number of days required for reentrainment were significantly increased in the 100 ng/*μ*l and 200 ng/*μ*l groups when compared with the PBS control group (two-way ANOVA, followed by Fisher's LSD post hoc test, *P* < 0.01). ^∗^*P* < 0.01.

The results of the wheel-running experiments showed that when the light/dark cycle was delayed by 8 h, the rd mice in the PBS control group (*n* = 3) were capable of reentraining to the light/dark cycle, and they took 4.67 ± 0.79 days to complete the synchronization with the shifted cycle (Figure 4(a)); while in the 100 ng/*μ*l group (*n* = 5) and the 200 ng/*μ*l group (*n* = 5), the mice were also able to reentrain but characterized by a delay, and they required 7.90 ± 0.55 days and 11.00 ± 0.79 days to complete the synchronization with the new light/dark cycle, respectively (Figures 4(b) and 4(c)). The differences in the number of days needed for reentrainment were statistically significant in all pairwise comparisons (all *P* < 0.01) (Figure 4(e)). In addition, the locomotor activity of the rd mice was less robust than that of the wild-type mice (Figure 4(d)). We also found that in comparison to the controls, the mice injected with immunotoxin were less able to precisely synchronize their activity to the shifted cycle, expressed as a significantly greater variability in activity onsets with respect to the beginning of the dark phase.

## 4. Discussion

It has been proven in many studies that ipRGCs are not only intrinsically photosensitive but also capable of transmitting the photic signals from rods and cones to the brain areas to mediate NIF functions. In order to clearly establish the roles of ipRGCs in NIF visual functions, ablation of the whole cell is required. In some recent studies, the whole cells of ipRGCs were destructed via specific binding to melanopsin. But another study revealed that there were at least 5 subtypes (M1–M5) of ipRGCs with distinct morphological and physiological characteristics; although being intrinsically photosensitive to drive photic reactions, some subtypes might have no or leaky expression of melanopsin or have transiently expressed melanopsin during development [10]. Therefore, the method of specific binding to melanopsin may be inadequate to ablate all subtypes of ipRGCs. In the presence of rods and cones, some subtypes of ipRGCs may still function to combine and convey the signals derived from the ONL; thus, we used the rd mice lacking rods and cones so as to evaluate the effect of partial ablation of ipRGCs on NIF functions in the absence of rods and cones.

Saporin (SAP) is a ribosome-inactivating protein of type I, which can irreversibly inhibit protein synthesis during transcription cycle. But binding of SAP to specific targets to enter into the cell is required for such inhibition. Goz et al. [23] developed a specific immunotoxin consisting of an antimelanopsin antibody conjugated to saporin, called melanopsin-SAP. Intravitreal injection of this immunotoxin could result in targeted destruction of ipRGCs in a dose-dependent manner, and this is the reason why we chose it for partial ablation of ipRGCs in our study. But this immunotoxin also has some limitations; as the targeted ablation of ipRGCs is also based on the binding of melanopsin-SAP to melanopsin, it raises the possibility that some nonmelanopsin-expressing subtypes of ipRGCs still survive after the injection of this immunotoxin. Thus, in our study, we used the rd mice lacking rods and cones to exclude the signals from photoreceptors.

Prior studies [23] reported that the loss of ipRGCs became stable at 3 weeks after intravitreal injection of melanopsin-SAP. So we decided to immunohistochemically label ipRGCs to count and compare the number of ipRGCs on flat-mounted retina at 1 month after the immunotoxin injection. Our findings revealed that the ipRGCs were partially ablated following intravitreal injection of immunotoxin melanopsin-SAP in rd mice. The survival rate of ipRGCs decreased from 75% to 40% as the melanopsin-SAP dose was increased from 100 ng/*μ*l to 400 ng/*μ*l, suggesting that the administration of melanopsin-SAP is associated with a dose-dependent targeted ablation of ipRGCs, at least in rd mice.

In order to verify whether all damages to ipRGCs were caused by the injection of melanopsin-SAP, we immunohistochemically labeled ipRGCs using antibodies against melanopsin and compared the number of ipRGCs on flat-mounted retina among 1-, 3-, and 6-month-old C3H/HeJ mice. We found that in adult C3H/HeJ mice, the number of ipRGCs did not alter with age, indicating that the injection of the immunotoxin induced all damages to ipRGCs.

We hypothesized that the injection of melanopsin-SAP would not result in major changes to the retinal structure other than a decrease in the number of ipRGCs. To test this, we had to assess the retinal conditions after the injection. Despite the availability of several measurement tools, the INL and ONL thickness is considered as a direct indicator reflecting the cellular state in each layer of the retina. Due to the almost complete loss of ONL in rd mice, we only measured and analyzed the INL thickness using IPP software. We did not observe any significant difference in the INL thickness between the highest dose (400 ng/*μ*l) group and the control group, suggesting that the exposure to melanopsin-SAP did not alter the morphology and structure of the retina. Since the highest dose of melanopsin-SAP did not result in any changes to the retinal structure, it is speculated that lower doses should not cause such changes, and thus, the INL thickness was not measured in other dose groups.

In our study, the effect of melanopsin-SAP on visual acuity was not assessed, because the rd mice used in this study had already lost nearly all of their rods and cones, thereby leading to almost complete visual loss.

As for the assessment of NIF visual functions, we focused on the reentrainment response, because it has been proven that this function is mainly regulated by ipRGCs [26]. Our study also demonstrated that the number of days needed for reentrainment was similar between rd mice (control group) and wild-type mice, indicating that rods and cones might play an insignificant role in this function.

After targeted destruction of ipRGCs via intravitreal injection of melanopsin-SAP, wheel-running experiments showed that the rd mice in the 100 ng/*μ*l dose group spent about 8 days to complete the synchronization with the new light/dark cycle and those in the 200 ng/*μ*l dose group required about 11 days to reentrain; the survival rates of ipRGCs were calculated to be 74.14% ± 4.15% and 39.25% ± 2.29% in the two groups, respectively. In comparison, the rd mice in the PBS control group and the wild-type mice only took around 4 days to resynchronize with the shifted cycle. These findings revealed that when the loss rate of ipRGCs reached about 25%, the reentrainment response of rd mice would be somewhat affected. More severe ipRGCs loss would result in a longer time needed to reentrain.

According to published literatures, a lower survival rate of ipRGCs was associated with a greater impairment of the reentrainment function in a cell number-dependent fashion, which was consistent with our findings (Table 1). Hatori et al. reported that, when the survival rate of ipRGCs was less than 10%, all animals seemed unable to reentrain [24]. In another study, with a survival rate of ipRGCs ranging from 18% to 40% after the injection of melanopsin-SAP, half of the animals failed to reentrain to the shifted light/dark cycle; the other half were capable of reentraining, but a longer time (more than 16 days) was required for such reentrainment [23]. In our study, when the survival rate of ipRGCs ranged from 40% to 75%, a greater number of days (at least 8–11 days) were needed for reentrainment as compared with the control group, though all animals were able to resynchronize with the new light/dark cycle. These results consistently indicate that the regulation of the reentrainment function is dependent on a certain minimal number of ipRGCs.

Taken together, we believe that a minimal density of ipRGCs is required to maintain the NIF visual functions. Similarly, a threshold relationship has also been identified between the lesion extent of the cholinergic basal forebrain (CBF) and working memory impairment in rats; only when the density of CBF was lower than 25%, impaired working memory could be observed [28]. Also, impairment of IF functions, such as visual field changes, could only be clinically detected when the loss rate of RGCs reached >40% [29]. The same is true for NIF functions; a recent study revealed that in patients with Leber hereditary optic neuropathy (LHON) and dominant optic atrophy (DOA), despite a moderate loss of ipRGCs, the NIF functions were well maintained including the photoentrainment of circadian rhythms, light-induced suppression of melatonin secretion, and pupillary light reflex [30]. This further supports that only when the loss of ipRGCs reaches a certain threshold can the NIF functions be affected.

Our study provides evidence that a lower survival rate of ipRGCs is associated with a greater impairment of the reentrainment function in a cell number-dependent manner. Further validation in larger animals and clinical patients (wrist watch for circadian rhythm monitoring is now clinically available) is still required, which may help to quantify such association and set standards for clinical examinations. For instance, our prior study and other published studies consistently showed that chronic ocular hypertension resulted in damages to ipRGCs and hence the reentrainment function [25, 31], indicating that we may assess the effect of glaucoma on ipRGCs by measuring the reentrainment response.

Moreover, this technique of specifically ablating melanopsin cells potentially has a wide range of applications, which may also be used in larger animals. A recent study in rat models also proved that melanopsin-SAP could specifically deplete ipRGCs in a dose-dependent fashion [32]. In order to obtain data more pertinent to humans, future studies should evaluate the effectiveness and specificity of this ipRGC immunotoxin in larger animal models where genetic modification seems inappropriate. This will help to provide more accurate and reliable information for understanding the mechanism of circadian rhythm-related disorders and offer insights into the potential treatment of these disorders.

In addition, as shown in Table 1, the experimental conditions varied among the published studies. Thus, we need to use a uniform design to optimize the experimental conditions including animal species, irradiance level, and intervention factors, so as to accurately define the association between the number of ipRGCs and NIF visual functions. Furthermore, as ipRGCs consist of several subtypes projecting to different brain areas, future studies may need to clearly establish the individual roles of each subtype in regulating NIF functions.

## Figures and Tables

**Table 1:** Effect of partial ablation of ipRGCs on reentrainment: comparison of study results.

	Hatori et al. [24]	Guler et al. [22]	Goz et al. [23]	Boudard et al. [27]	Our findings
Reentrainment	Absent (150 lx)	Most animals unable to entrain (700 lx) Advanced onset Others had light responses, but no stable circadian rhythms	Half able to entrain, more than 16 days required (100 lx) Advanced onset Half failed to entrain	Able to entrain, more than 12 days required (15 lx) Normal (300 lx)	Able to entrain, at least 8–11 days required (100 lx) Advanced onset
Survival rate	<10%	3–17%	18–40%	63%	40–75%
